# Population prevalence of hereditary breast cancer phenotypes and implementation of a genetic cancer risk assessment program in southern Brazil

**DOI:** 10.1590/S1415-47572009005000058

**Published:** 2009-09-01

**Authors:** Edenir I. Palmero, Maira Caleffi, Lavínia Schüler-Faccini, Fernanda L. Roth, Luciane Kalakun, Cristina Brinkmann Oliveira Netto, Giovana Skonieski, Juliana Giacomazzi, Bernadete Weber, Roberto Giugliani, Suzi A. Camey, Patricia Ashton-Prolla

**Affiliations:** Programa de Pós-Graduação em Genética e Biologia Molecular, Universidade Federal do Rio Grande do Sul, Porto Alegre, RSBrazil; 2 Laboratório de Medicina Genômica, Hospital de Clínicas de Porto Alegre, Porto Alegre, RSBrazil; 3 Núcleo Mama Porto Alegre and Associação Hospitalar Moinhos de Vento, Porto Alegre, RSBrazil; 4 Serviço de Genética Médica, Hospital de Clínicas de Porto Alegre, Porto Alegre, RSBrazil; 5 Departamento de Genética, Universidade Federal do Rio Grande do Sul, Porto Alegre, RSBrazil; 6 Instituto Nacional de Genética Médica Populacional, Porto Alegre, RSBrazil; 7 Programa de Pós-Graduação em Ciências Médicas, Universidade Federal do Rio Grande do Sul, Porto Alegre, RSBrazil; 8 Programa de Pós-Graduação em Epidemiologia, Universidade Federal do Rio Grande do Sul, Porto Alegre, RSBrazil; 9 Departamento de Estatística, Instituto de Matemática, Universidade Federal do Rio Grande do Sul, Porto Alegre, RSBrazil

**Keywords:** breast cancer, genetic counseling, hereditary cancer syndromes

## Abstract

In 2004, a population-based cohort (the Núcleo Mama Porto Alegre - NMPOA Cohort) was started in Porto Alegre, southern Brazil and within that cohort, a hereditary breast cancer study was initiated, aiming to determine the prevalence of hereditary breast cancer phenotypes and evaluate acceptance of a genetic cancer risk assessment (GCRA) program. Women from that cohort who reported a positive family history of cancer were referred to GCRA. Of the 9218 women enrolled, 1286 (13.9%) reported a family history of cancer. Of the 902 women who attended GCRA, 55 (8%) had an estimated lifetime risk of breast cancer ≥ 20% and 214 (23.7%) had pedigrees suggestive of a breast cancer predisposition syndrome; an unexpectedly high number of these fulfilled criteria for Li-Fraumeni-*like* syndrome (122 families, 66.7%). The overall prevalence of a hereditary breast cancer phenotype was 6.2% (95%CI: 5.67-6.65). These findings identified a problem of significant magnitude in the region and indicate that genetic cancer risk evaluation should be undertaken in a considerable proportion of the women from this community. The large proportion of women who attended GCRA (72.3%) indicates that the program was well-accepted by the community, regardless of the potential cultural, economic and social barriers.

## Introduction

Latin America is formed by low-to-medium income countries with health indicators that are evolving towards patterns seen in developed nations. The extensive ethnic and cultural diversity seen in these countries reflects their history and different degrees of admixture between native and immigrant populations. Brazil is the largest and most populated country in Latin America, with ~170 million inhabitants. Although Brazil has the eighth largest gross national product in the world, the average per capita income ranks only 39^th^. A significant challenge in Brazil and other Latin American countries is the inclusion of new health technologies, including genetic risk assessment and testing, in health care systems that have significant budget constraints.

The Brazilian constitution guarantees the right to medical assistance to every citizen and at least 75% of the population depends almost entirely on health care provided by the government (SUS - *Sistema Único de Saúde*). One special SUS program designed for community-based health care is the *Programa Saúde da Família* (PSF or Family Health program), created in the mid-90s and based upon multidisciplinary teams composed of a physician, nurse, 1-2 nursing assistants and 4-6 lay community health workers. Each team provides primary health care for a geographically defined group of approximately 600 families. The Family Health program has expanded rapidly and currently provides health care to about half of the population (Ramalho and Silva, 2000; Brasil, Ministério da Saúde, 2003a, 2003b; Harzheim *et al.*, 2006).

With few exceptions, departments of health at city, state and national levels do not have explicit policies for the prevention and care of people with genetic disorders. Genetic services are mainly centered in teaching hospitals of major cities and cancer genetic testing *per se* is not yet covered by SUS or private health insurance. In Porto Alegre, the two existing public GCRA services are located in tertiary care centers (Penchaszadeh, 2000; Llerena, 2002; Palmero *et al.*, 2007a).

Breast cancer is a significant public health problem throughout Brazil, and is currently the first cause of cancer-related deaths in Brazilian women of all ages. Rio Grande do Sul (RS), Brazil's southernmost state, has one of the highest incidences of breast cancer in the country and an increasing breast cancer mortality rate, despite efforts to improve the coverage for breast cancer screening (Brazil, 2003b). Breast cancer is also the leading cause of death by cancer in young women (30-49 years) from this region (Cadaval Gonçalves *et al.*, 2007). The estimated incidence for breast cancer in RS and Brazil in 2008 was 85.5 and 51.2 per 100,000, respectively (Brazilian National Cancer Institute, 2008; Brasil, Ministério da Saúde, 2003b). Recently, SUS has anticipated the recommended age at initiation for annual mammographic screening from 50 to 40 years. However, routine mammographic screening is not yet readily available.

One of the most important risk factors for breast cancer is a positive family history, and an estimated 5%-10% of all diagnosed cases is hereditary, *i.e.*, caused by germline mutations in high penetrance predisposition genes (De la Chapelle and Peltromaki, 1998; Offit, 1998; Margolin and Lindblom, 2006). Germline mutations in the breast cancer genes *BRCA1* and *BRCA2* are related to an increased risk for breast, ovarian and other cancers in a syndrome known as hereditary breast and ovarian cancer (HBOC) which accounts for most cases of hereditary breast cancer (HBC) worldwide (Miki *et al.*, 1994; Wooster *et al.*, 1994; Peto *et al.*, 1999; Anglian Breast Cancer Study Group, 2000; Antoniou *et al.*, 2000, 2002). Other genes that predispose to breast cancer, such as *TP53* (associated with Li-Fraumeni and Li-Fraumeni-*like* syndromes, LFS/LFL) (Li and Fraumeni, 1969; Birch *et al.*, 1994; Eeles, 1995) *PTEN* (associated with Cowden's syndrome) (Eng, 1997) and *CHEK2* (associated with hereditary breast and colon cancer syndrome, HBCC) (Meijers-Heijboer *et al.*, 2003) have been identified and are thought to have important, albeit lower, contributions to the phenotype (Li *et al.*, 1988; Vahteristo *et al.*, 2002). The identification of individuals with these syndromes is important to ensure that appropriate strategies to reduce the risk of cancer are recommended to these persons and their families (Nelson *et al.*, 2005; Guillem *et al.*, 2006).

Little attention has been given to the identification and study of hereditary breast cancer phenotypes in the community and primary health care services (De Silva *et al.*, 1995; Pharoah *et al.*, 2000; Hall *et al.*, 2001; Hoskins *et al.*, 2006). In this context, the main purpose of this study was to assess the prevalence of a significant family history of cancer and of hereditary breast cancer phenotypes in an underserved community with high breast cancer incidence and mortality rates in southern Brazil.

## Subjects and Methods 

In April 2004, a large population-based cohort study (the Núcleo Mama Porto Alegre - NMPOA Cohort) was started in Porto Alegre, the capital of the southern Brazilian state of Rio Grande do Sul. The cohort intends to collect demographic, epidemiologic and risk factor data from a large sample of women 15 years old and test a model for community-based breast cancer screening for women between the ages of 40 and 69 years, as described elsewhere (Caleffi *et al.*, 2009 Smith *et al.*, 2006). Women > 15 years old who visited primary health care units as part of the Family Health program in seven underserved areas of Porto Alegre were included in the NMPOA cohort from April 2004 through March 2006.

###  Patient recruitment

The family history of breast cancer and other tumors was assessed in all of the patients included in the NMPOA cohort by using a seven question instrument (Table 1) and considered both maternal and paternal histories in first-, second- and third-degree relatives. The instrument has been validated for this population (manuscript submitted) (Ashton-Prolla P, Giacomazzi J, Schmidt AV, Roth FL, Palmero EI, Kalakun L, Aguiar E, Moreira SM, Batassini E, Belo-Reyes V, Caleffi M, Camey S; unpublished data) and was based on features associated with an increased likelihood of clinically significant *BRCA* mutations (Couch *et al.*, 1997; Shattuck-Eidens *et al.*, 1997; Srivastava *et al.*, 2001; Frank *et al.*, 2002; Nelson *et al.*, 2005). Furthermore, a question about the family history of breast cancer and/or colon cancer was included because of previous evidence indicating a higher than expected prevalence of such an association based on patients followed at cancer genetics clinics in Porto Alegre (Palmero *et al.*, 2007b).

Women above the age of 18 years old who replied positively to at least one of the questions at the primary health care unit were referred for GCRA and invited to participate in this study. Ethical approval (Protocol number 04-170, coordinating center IRB, Hospital de Clínicas de Porto Alegre) was obtained from the institutions involved and enrollment in the study required signature of informed consent. Active recruitment was initiated after six months if the patients referred by the Family Health program did not reach NMPOA. In this case, three attempts were made to schedule a visit by telephone, followed by a letter of invitation and a search for the patient by community agents. If all of these strategies failed, or if three scheduled appointments were not kept, no further contact was attempted.

###  Patient sample for weighted prevalence analysis

To determine the weighted prevalence of a hereditary breast cancer phenotype in the population being studied, two groups of patients were evaluated: (a) 885 unrelated women with a family history of cancer and (b) 910 unrelated women of the same cohort with no family history of cancer upon recruitment at the primary health care unit and who were invited to participate in this study during their annual mammographic screening examinations. Recruitment was done consecutively during a period of 12 months. The evaluation included an interview, an estimation of the risk of breast cancer, and registration of the family history in pedigrees of at least three generations. The presence of criteria for breast cancer predisposition syndromes was assessed by three clinical geneticists (PAP, FLR and CBON) who independently reviewed each pedigree. The group of patients referred for genetic risk evaluation was also the group used for the validation of the seven question instrument for identification of hereditary breast cancer families (Ashton-Prolla P, Giacomazzi J, Schmidt AV, Roth FL, Palmero EI, Kalakun L, Aguiar E, Moreira SM, Batassini E, Belo-Reyes V, Caleffi M, Camey S; unpublished data).

###  Genetic cancer risk assessment (GCRA)

Genetic evaluation included recording of the medical and family histories of each index case in detailed pedigrees, with information traced as far back (minimum of three generations) and laterally as possible and including paternal lineages. Confirmation of the family history of cancer was attempted in all cases and pathology reports, medical records and/or death certificates were obtained whenever possible (unpublished data). Estimated lifetime risks of breast cancer (ELTR) were obtained by using Claus tables and the Gail and Tyrer-Cuzick models (Gail *et al.*, 1989; Claus *et al.*, 1994; Domchek *et al.*, 2003; Tyrer *et al.*, 2005). The clinical diagnosis of Hereditary Breast and Ovarian Cancer (HBOC) Syndrome was based on the criteria of the American Society of Clinical Oncology (ASCO, 1996; Ford *et al.*, 1998). In addition, the prior probabilities of carrying a *BRCA1* or *BRCA2* mutation were determined for each patient by using mutation prevalence tables and a modified Couch (Penn II) mutation probability model (Frank *et al.*, 2002; Domchek *et al.*, 2003, 2004). All of the pedigrees were reviewed by at least two clinical geneticists to assess the presence of criteria for LFS, LFL, HBCC or other cancer predisposition syndromes. For LFS, LFL and HBCC, previously published criteria for their clinical diagnosis were used (Li and Fraumeni, 1969; Birch *et al.*, 1994; Eeles, 1995; Meijers-Heijboer *et al.*, 2003). Three breast cancer risk categories were established, namely, Risk I: < 0.2 using all three models of ELTR, Risk II: ≥ 0.2 with at least one of the models, and Risk III: women with a family history that fulfilled the criteria for a breast cancer predisposition syndrome.

###  Follow up

All of the patients were encouraged to perform monthly breast self-examinations. Women with ELTR < 0.2 (Risk I) were referred back to their primary health care unit for prospective follow-up as determined by their age and other non-genetic risk factors (monthly breast self-examination, annual clinical exam and annual mammography for women 40-69 years old). Patients with ELTR ≥ 0.2 (Risk II) and those who fulfilled criteria for hereditary breast cancer syndrome (Risk III) were referred for clinical breast evaluations at 6-month intervals. In addition, for women in risk categories II and III, a recommendation was made for annual mammography intercalated with breast magnetic resonance imaging at 6-month intervals; breast ultrasound was recommended for those with very dense breasts and/or whenever clinically indicated. Women in risk category III began screening at the age of 25 years or as soon as a risk was identified. Those with an increased risk for tumors other than breast cancer were referred to tertiary care centers for inclusion in comprehensive cancer screening programs. Those with criteria for a breast cancer predisposition syndrome were offered genetic testing and the results of this investigation will be described elsewhere.

After conclusion of the genetic evaluation, written reports and a brochure with key information about the prevalence, treatment and prevention of breast cancer, as well as information about hereditary breast cancer, were mailed to all of the patients. The vast majority of the women included in this study relied exclusively upon the public health care system. Although mammographic screening and clinical follow-up are available in this system outside the cohort study described here, access to these facilities is limited and most women would not have been able to comply with the recommended screening guidelines if they had not been included in this study. Magnetic resonance imaging of the breast is not routinely available in the public health care system.

###  Statistical analysis

Quantitative variables were expressed as the mean ± SD, whereas categorical variables (descriptive analyses) were recorded as absolute and/or relative frequencies. The χ^2^ test was used to compare the distribution of the answers to the family history questionnaire between attenders and non-attenders, whereas ANOVA was used to compare the mean values of the risk estimates and the history of breast cancer among the different risk categories. Kappa coefficients were used to assess the agreement between results provided by the instrument and the estimated genetic cancer risk. The weighted prevalence of hereditary breast cancer phenotypes was calculated using the following weighting: group with a family history of cancer (885/1285) and group with no family history of cancer (910/7933). All data handling and statistical analyses were done using the statistical software package SPSS (version 14.0), and a value of p < 0.05 indicated significance.

## Results

###  Description of the population served by the program

The women enrolled in this study were residents of seven regions in Porto Alegre. These regions correspond to 64% of the total area of the city and ~35% of its population. All women relied almost exclusively on the Family Health Program for health care. Although specific demographic data for this population of the Family Health Program clients was unavailable, data from the most recent municipal census indicate that 49.2% of the inhabitants in these regions have < 8 years of education (5.6% are illiterate), 20% live in temporary homes and 6.5% of family providers have no income (Porto Alegre, Prefeitura Municipal 2004).

###  Patient recruitment

**Figure 1:**
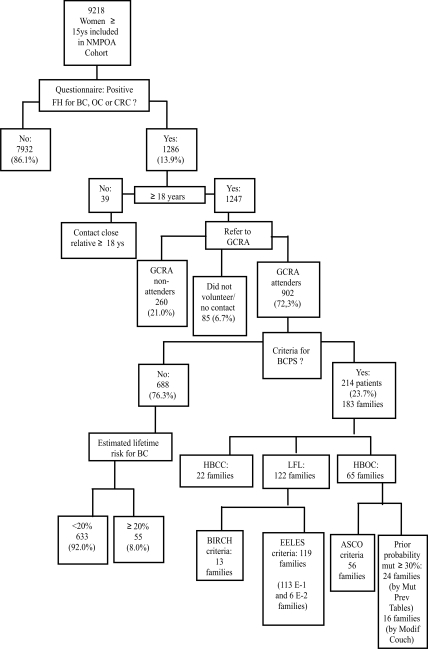
Preliminary results of genetic cancer risk assessment in a population-based cohort of women > 15 years of age in Porto Alegre, Brazil. GCRA – genetic cancer risk assessment; FH – family history; BC – breast cancer; OC – ovarian cancer; CRC – colorectal cancer; BCPS – breast cancer predisposition syndrome; HBCC – hereditary breast and colorectal cancer syndrome; LFL – Li-Fraumeni-like syndrome; HBOC – hereditary breast and ovarian cancer syndrome.

Of the 9218 women enrolled in the cohort, 1286 (13.9%) answered positively to at least one of the seven questions about a family history of cancer (Figure 1) and those above the age of 18 years old (n = 1247) were referred for GCRA. Of these, 260 (21%) did not reach NMPOA and did not respond to the active recruitment strategies within a 12 month period after inclusion in the cohort. In addition, 41 (3.3%) women scheduled an appointment three times but did not show up, and another 43 (3.4%) did not wish to participate in the study. The remaining 902 women from 829 families were compliant with the referral and attended GCRA.

The demographic data of women with a positive family history who underwent GCRA (attenders) differed significantly in some aspects from those of women who did not undergo GCRA (non-attenders). Non-attenders were generally younger, less educated, and had undergone a breast biopsy less often than attenders (p < 0.001). For most of the questions in the family history questionnaire, there was no significant difference between the proportion of positive answers between attenders and non-attenders. Exceptions to this included: (a) the question about a family history of breast and/or ovarian cancer in ≥ 2 relatives, for which attenders provided a greater number of positive answers, and (b) the question about the combined occurrence of breast *and* ovarian cancer in a relative, for which non-attenders provided a greater number of positive answers (Table 1). The latter observation may reflect a bias of ascertainment with consequent deficiency in providing GCRA to some individuals with a high risk of HBOC. However, when the answers to this question (family member with multiple tumors) at the primary health care unit were compared to the family history reported by attenders during GCRA, and confirmed by review of medical records, there was low agreement between the responses observed, *i.e.* only one diagnosis (usually breast cancer) remained in most cases (Kappa coefficient = 0.069).

###  Risk assessment

Table 2 summarizes the demographic and other variables related to the estimation of lifetime risk for developing breast cancer in the 902 attenders. A significant proportion of the women were smokers and overweight or obese, as also observed in the NMPOA cohort as a whole (Caleffi *et al.*, 2009). A high number of patients reported a positive family history for cancer in the maternal lineage: 26.1% had a family history of breast cancer only, whereas 19.8% had a family history of breast cancer and colorectal cancer (CRC). Most of the women evaluated (688, 76.3%) did not fulfill criteria for breast cancer predisposition syndromes. Among these women, the estimated lifetime risk of developing breast cancer was < 0.2 in 633 (92%) and ≥ 0.2 in 55 (8%). The most common feature of the family history that justified GCRA in this group of patients was the presence of a relative with breast cancer who was < 50 years old (Table 3). The remaining 214 women (23.7%) from 183 families had pedigrees suggestive of breast cancer predisposition syndrome. Of these 183 families, the majority fulfilled criteria for the Li-Fraumeni-*like* syndrome (Figure 1). The overall weighted prevalence of a breast cancer predisposition syndrome phenotype in the sample studied was 6.2%. Twenty-five families fulfilled criteria for more than one syndrome. The 76 patients (65 families) who fulfilled criteria for HBOC syndrome had average *BRCA* mutation probabilities of 21.9 ± 13.9% and 25.7 ± 14.8% by the Penn II model and *BRCA* mutation prevalence tables, respectively. For all of the attenders who were unaffected by cancer, the ELTR estimates obtained using the Claus tables and the Gail and Tyrer-Cuzick models were 13.6%, 13.9% and 13.9%, respectively. Table 3 summarizes the family history of breast cancer and ELTR data for patients in the three risk categories.

## Discussion

By using a simple seven-question instrument to inquire about the family history of cancer in women from primary health care units of an underserved region in southern Brazil, we identified a significant proportion with family histories of cancer who fulfilled criteria for one of the most commonly recognized hereditary breast cancer syndromes. The overall prevalence (6.2%) was similar to that described in other population-based samples, although only a few studies have addressed this issue to date (Pharoah *et al.*, 2000; Hughes *et al.*, 2003; Palomaki *et al.*, 2006). Most of the families with hereditary breast cancer phenotypes corresponded to LFL syndrome. Although there is a phenotypic overlap among the HBOC, LFS/LFL and HBCC syndromes, the high frequency of LFL pedigrees observed here is striking, especially considering that the original questionnaire used to identify these patients was not designed to screen for LFS/LFL.

Already ten years ago (Varley *et al.*, 1999) described certain low penetrance *TP53* alleles and suggested that deleterious mutations in *TP53* (or related genes) may be more frequent in the population than previously estimated. In addition, several recent reports have indicated that a deleterious germline mutation in *TP53*, R337H, might be very prevalent and related to a founder effect in southern Brazil (Pinto *et al.*, 2004; Achatz *et al.*., 2007; Palmero *et al.*, 2008). Although some of the criteria used here to diagnose the LFL phenotype were not stringent (*i.e.* Eeles criteria used to classify LFL phenotype) and may have little sensitivity for identifying germline *TP53* mutations in other countries, the unexpectedly high number of criteria-positive patients suggests that LFL may indeed be a common phenotype for hereditary breast cancer in and around Porto Alegre. Future results from germline mutation testing should help to clarify this issue and contribute to our understanding of the applicability and discriminatory capacity of breast cancer risk estimation models, current diagnostic criteria and mutation prediction models for hereditary breast cancer syndromes in this population. If such a high prevalence of LFS/LFL syndrome is confirmed among families in this geographic area, an effort will have to be made to facilitate the identification of high-risk individuals and delineate effective cancer screening and prevention programs in these subjects.

The relatively large proportion of women (72.3%) who attended the proposed GCRA was very encouraging and comparable to that encountered in similar studies, *e.g.*, 88% and 70% in underserved communities in the U.S.A. (Ricker *et al.*, 2006) and Singapore (Chin *et al.*, 2005), respectively. Furthermore, in a recent study by ONeill *et al.* (2006) in the U.S.A., the outcome of genetics referrals was evaluated in a group of women with estimated *BRCA* mutation probabilities ≥ 10%. Within six-months after referral, 36% of the patients had undergone genetic evaluations (acceptors), 27% still intended to seek (intenders) and 36% refused such assessment (decliners). Population-based mammographic screening programs worldwide have also reported compliance rates of 61%-83% (Banks *et al.*, 2002; O'Malley *et al.*, 2002; Finney *et al.*, 2006).

In this study, the number of attenders also has to be interpreted in light of the difficulties that most of these women face to seek advice, health care and cancer prevention opportunities. First, there might be a cultural difference in cancer-associated risk perception and/or a difficulty in understanding the impact of preventive interventions. The way of dealing with risk is not only influenced by risk perception but also by culture. This was clearly demonstrated by Hofstede (1997) in a landmark study in which different cultures around the world were characterized based on five parameters, including the uncertainty avoidance index (UAI). This index, which reflects the tolerability of a given society towards uncertainty and ambiguity, is higher in Brazil than in Denmark, the United Kingdom and the U.S.A. Such differences could interfere with cancer risk perception and motivation to seek GCRA. In addition, certain cultures are more fatalistic about cancer and perceive fewer benefits from screening (Russell *et al.*, 2006). Second, there may be a knowledge barrier in understanding how preventive measures will ultimately increase quality of life (Achat *et al.*, 2005; Ricker *et al.*, 2006; Farmer *et al.*, 2007). Third, in many of these women, care of themselves is often set aside because of more urgent needs, such as providing food, housing and education for their families. Fourth, it may be that these women are simply obeying and respecting a hierarchical structure in which they attend GCRA only because they are told to do so, and their cultural conditioning generally makes them follow health recommendations once they are given. Finally, there has been a historical lack of resources to ensure that adequate screening is provided in this area, even if there is evidence for a higher risk (Smith *et al.*, 2006). Consequently, most of these women may have seen the program offered here as an opportunity to be grasped since they would otherwise face significant difficulties in following standard recommendations for breast cancer screening if they relied solely upon the public health care system.

For all of these reasons, programs such as that described here, which attempt to identify and prospectively follow women with an increased risk of cancer, need to consider the importance of patient education and social interventions (*i.e.*, facilitate transportation, nutrition and childcare) in the difficult task of maintaining compliance to the recommended guidelines. A more detailed study of non-attenders may provide better clues on how to improve coverage for programs such as these, thereby ensuring that most high-risk patients have access to the information and preventive interventions they require.

In conclusion, the implementation of a GCRA program for an underserved community in southern Brazil revealed that the overall prevalence of a hereditary breast cancer phenotype in this population-based sample of women was 6.2%, which may be a matter of considerable concern in this region. In addition, the establishment of breast cancer risk categories allowed the identification of higher risk women who may benefit from more intensive screening. The relatively high number of women who attended GCRA sessions after an initial referral suggested that the program was well accepted by the community and is feasible, regardless of potential cultural, economic and social barriers.

## Figures and Tables

**Table 1:** Positive responses to the family history questionnaire given by 1,247 women referred to genetic cancer risk assessment (GCRA).

Question	GCRA non-attenders (n = 345)			GCRA attenders (n = 902)		p
	N	%		N	%	
Did any of your first degree relatives have breast or ovarian cancer?	122	35.4		378	42.0	0.118
Did any of your relatives have bilateral breast cancer?	48	14.1		112	12.4	0.561
Did any man in your family have breast cancer?	6	1.7		11	1.2	0.590
Did any woman in your family have breast *and* ovarian cancer?	47	13.9		44	4.9	< 0.001
Did any woman in your family have breast cancer before the age of 50 years?	214	62.4		568	63.0	0.551
Do you have two or more relatives with breast and/or ovarian cancer?	63	18.3		226	25.1	0.016
Do you have two or more relatives with breast and/or bowel cancer?	69	20.2		234	25.9	0.062

**Table 2:** Demographic data and characteristics of the 902 women who underwent genetic cancer risk assessment (GCRA)

	N	%	Mean	SD
Age at assessment (yr)	-	-	43.2	12.7
BMI			27.9	5.8
≤ 18.5	6	0.7	-	-
18.51-25	300	33.3	-	-
25.01-30	298	33.0	-	-
> 30	285	31.6	-	-
Smoking	262	29.0	-	-
Age at menarche	-	-	12.7	1.7
Parity				
No children	108	12.0	-	-
One or more children	790	88.0	-	-
Age at birth of first child	-	-	21.5	5.0
Reproductive status				
Pre-menopausal	585	65.2	-	-
Post-menopausal	312	34.8	-	-
Age at menopause	-	-	47.0	5.4
Endogenous hormone exposure (yr)	-	-	27.3	9.7
Hormone replacement therapy	73	8.1	-	
Consanguinity*	65	7.3	-	
Family history of cancer				
Side of family				
Maternal	554	62.7	-	-
Paternal	223	25.2	-	-
Maternal and paternal	58	6.6	-	-
Others (siblings/offspring)	49	5.5	-	-
Breast cancer family history				
Breast cancer only	234	26.1	-	-
Breast and ovarian cancer	87	9.6	-	-
Breast and colon cancer	179	19.8	-	-

BMI = body mass index; yr = years. The number of respondents varied because of missing information for some variables. *Evidence of consanguinity within the family, regardless of the relationship to the proband.

**Table 3:** Risk estimates and breast cancer history according to risk category in the 902 women who underwent genetic risk assessment.

	Estimated lifetime risk for breast cancer < 0.2 (n = 633)	Estimated lifetime risk for breast cancer ≥ 0.2 (n = 55)	Phenotype of breast cancer predisposition syndrome (n = 214)	p
Number of BC cases in family*	0.98 ± 0.67	1.45 ± 0.83	1.69 ± 1.14	< 0.001
Number of BC-affected generations*	0.92 ± 0.54	1.24 ± 0.55	1.29 ± 0.64	< 0.001
Average age (yr) at BC diagnosis in the family	46.6 ± 10.6	47.0 ± 11.4	46.6 ± 11.2	0.968
ELTR for BC				
Using the Gail model**	10.2 ± 4.1	19.2 ± 5.1	12.3 ± 6.6	< 0.001
Using the Claus model*	10.2 ± 2.8	16.7 ± 7.8	13.9 ± 7.4	< 0.001
Using the Tyrer-Cuzick model**	9.8 ± 3.7	19.6 ± 6.2	12.4 ± 5.6	< 0.001
Prior probability of mutation in a *BRCA* gene				
Mutation prevalence tables***	6.3 ± 3.8	6.7 ± 3.9	13.2 ± 13.0	< 0.001
Modified Couch model***	9.7 ± 4.3	10.3 ± 5.0	14.8 ± 10.6	< 0.001

The values are the mean ± SD. BC = breast cancer, BCPS = breast cancer predisposition syndrome, ELTR = estimated lifetime risk. *The mean value of the group with a slightly increased risk was significantly lower than that of the other two groups. **The mean values in all three groups differed significantly from each other. ***The mean value of the group that had criteria for breast cancer predisposition syndrome was significantly greater than that of the other two groups. Note: The number of valid cases used in each of the ELTR and prior probability analyses was 878, 592 and 874 for the Gail, Claus and Tyrer-Cuzick models, respectively. For the mutation prevalence tables and the modified Couch model, 890 and 874 valid cases were used, respectively.
